# Dr. Weatherhead on the Waters of Leamington

**Published:** 1821-06-01

**Authors:** 


*An Analysis of the Leamington Spa, in Warwickshire; with Remarks on its Use, and medicinal Qualities.* By G. H. Weatherhead, M. D. Graduate of the University of Edinburgh, Member of the Royal College of Physicians, &c. Second Edition, 8vo, sewed, pp. 45. London, 1820.

The author appears to have enrolled himself among the tutelar divinities, which, according to Pliny, were supposed to guard medicinal springs and contribute to the happiness of mankind, as his motto pretty evidently insinuates.

> " Publica morborum requies, commune medentem
> " Auxilium, PRÆSENS NUMEN, inempta salus."

The writer of this pamphlet appears also to be a man of great and universal erudition, for indications of science and literature are profusely poured forth in every page, and even upon the most common occasions. Thus, instead of informing his readers that the Leam is slow in its course, because the ground is nearly level, he learnedly pronounces it to be —" *torpid* in its course, from want of sufficient *descent*," which has a far greater air of classical elegance. " Leamington is a place of some antiquity, and retains the *agnomen* of priors from its being formerly attached to the monastery of Kenilworth."

Independently of the health-restoring waters of Leamington, numerous other attractions are here pourtrayed for the visiter. " If romance be the *gusto*, Guy's Cliff and Warwick Castle present themselves." " To the *pilgrim of genius*, Stratford upon Avon, the birth-place of the immortal Shakespeare, affords ample scope to indulge his warmest enthusiasm." P. 9.

We shall pass over our author's geological remarks, and also his chemical examination of the waters, since we find that Dr. Scudamore and he do not agree in their results. We have also stated enough in our last number, respecting the composition of these springs. " With regard to the iron which the waters *testify* on the addition of tincture of galls," our author thinks it owing to the iron pipes through which they flow; and as to what is called the chalybeate spring, Dr. Weatherhead considers it to have no claim to that appellation. "Indeed it appears to be simply a brackish water, with a very slight impregnation of the usual saline constituents." 24.

*Medicinal Properties.* These waters are considered by our author as aperient and diuretic; their laxative operation being gentle, unaccompanied by griping, and leaving no relaxed or exhausted feeling afterwards. There is no debility, he avers, produced by their use, even if continued daily for three weeks together. On the contrary, they increase the appetite, clear the complexion, and invigorate the whole system. The principal diseases to which these waters are more particularly applicable, are ophthalmia, dispeptic affections, liver complaints, chronic dysentery, jaundice, scrofula, cutaneous complaints, paralytic affections, diseases of the joints, rickets, debility, and irritability.

The usual and best time, Dr. W. observes, for drinking the waters, is before breakfast. The common dose is a tumbler full for an adult, which is to be repeated in a quarter of an hour, the patient walking about in the interim. The walk is to be continued for a short time after the second dose, when tea is to be drunk freely for breakfast. Where the usual dose of the spring is too weak to produce its operation, a blue pill is sometimes taken over night—a plan which Dr. W. cannot approve of, as he thinks it better to quicken the action of the water by adding some of the crystallized salt. Not being one of the initiated in the mysteries of the place, we cannot pretend to dispute the point; but from what we have seen of visceral disease, and from what we know of the practice of able physicians at similar watering places, we should not be disposed to condemn the blue pill in conjunction with deobstruent waters.

In respect to dietetics, our author's remarks are of the most common-place kind; and although he can so liberally criticise the introduction of poetical quotations into the writings of others, he hesitates not to press them, on all occasions, into his own service! And here we shall close this short article, with an observation directed to nobody, unless somebody should consider it as not inapplicable to his own case. It is this: that when a medical writer seats himself in the critic's chair, he should not "travel beyond his RECORD," and, under pretence of passing judgment on *books*, give vent to the most scurrilous abuse of *men*. Such conduct is now readily appreciated by the profession, and rewarded accordingly.

